# Effects of Rearing Density on Survival, Growth, and Development of the Ladybird *Coleomegilla maculata* in Culture

**DOI:** 10.3390/insects6040858

**Published:** 2015-10-09

**Authors:** Eric W. Riddick, Zhixin Wu

**Affiliations:** National Biological Control Laboratory, Jamie Whitten Delta States Research Center, ARS-USDA, Stoneville, MS 38776, USA; E-Mail: zhixin.wu@ars.usda.gov

**Keywords:** Coccinellidae, brine shrimp, lady beetle, mass production, pest management

## Abstract

Our research focuses on developing techniques to rear ladybird beetles (Coleoptera: Coccinellidae). We evaluated the effects of rearing density on survival, growth, and development of *Coleomegilla maculata*. The hypothesis that a low to moderate rearing density has limited or no effects on survival and development was tested. *C. maculata* first instars were reared to pupae at a density of 1, 5, 10, 15, or 20 individuals per arena (2.5 cm high, 9.0 cm diameter, and 159 cm^3^ volume) and fed powdered brine shrimp (*Artemia franciscana*) eggs. More larvae survived at the 1 and 5 densities, but no differences were detected between the 10, 15, or 20 densities. Median survival rate was at least 90% for larvae and 100% for pupae at the 10, 15, and 20 densities. Development time, body weight, and sex ratio were unaffected by rearing density. Overall, this study suggests that *C. maculata* larvae can be reared successfully at a density of 20 larvae/159 cm^3^ (≈ 0.126 larvae/cm^3^) in containers provisioned with powdered *A. franciscana* eggs. Scaling-up the size of containers, and *C. maculata* density in these containers, should be possible.

## 1. Introduction

*Coleomegilla maculata* (*C. maculata*) DeGeer (Coleoptera: Coccinellidae) is a native ladybird beetle distributed in ecosystems in North, Central, and South America [1,2,3,4]. It is polyphagous, consuming eggs and young larval stages of many small, soft-bodied insects and mites, but shows a feeding preference for aphids [5,6,7] and plant pollen [8,9]. There is interest in using this ladybird beetle to control pests in field crops [10,11,12], to test non-target effects of plant toxins [13], and to control pests in protected culture, *i.e.*, high tunnels, greenhouses, plantscapes and interiorscapes [14,15,16].

A common approach to controlling pests in protected culture, using predators (e.g., ladybird beetles), is to inundate infested plants with immature or adult stages. This necessitates a mass rearing program. For cost effective mass rearing, conservation of space in rearing facilities is essential. A first step to conserving space is to maximize insect density in rearing containers, without negatively affecting the quality of reared individuals. Rearing multiple predators in the same container can cause a change in the behavior of individuals, and potentially promulgate harmful effects on survival, development, and growth [17]. Some coccinellid larvae cannibalize their cohorts (siblings and non-siblings) under crowded conditions, especially if the food source is not suitable in quality or quantity [18,19,20]. For some species, larval-larval cannibalism occurs despite the availability of high quality food; consequently, researchers rear larvae individually rather than in groups [21]. Curbing or eliminating cannibalistic behavior so that larvae can be reared in groups is necessary for efficient mass production [22,23]. Therefore, the capacity to increase the density of immature predators within rearing systems, while eliminating or limiting cannibalism is a grand challenge to space-efficient mass production. A mass rearing system has no hope of becoming cost effective, unless space saving methods, such as group rearing of predators, are implemented.

Increasing rearing density without decreasing pre-imaginal survival and development are major obstacles to mass rearing coccinellids [24,25,26], anthocorids [27] and reduviids [28,29]. Provisioning rearing containers with refuges (*i.e.*, hiding places) can improve the capacity to increase rearing density, with varying degrees of success for coccinellids [30,31] and anthocorids [32]. Clearly, more research on this topic is necessary, particularly to support efforts to develop rearing systems useful for the biocontrol industry (natural enemy producers). The impetus of our ongoing research has been to develop a cost- and space-efficient rearing system for *C. maculata*. The purpose of this study was to determine the effects of rearing density on growth, development, and survival of *C. maculata* in the laboratory. In our recent works [14,15,16], we designed experiments to test the suitability of alternative foods for *C. maculata* larvae, reared in groups of 10–12 larvae per arena. Many of our critics claimed that rearing this species in such groups, even when the food source was deemed suitable, was inappropriate. Hence, this study also serves to confirm our previously published reports and show that group rearing is possible. The hypothesis that a low to moderate rearing density has limited or no effects on pre-imaginal survival and development was tested.

## 2. Experimental Section

### 2.1. Research Subjects and Food Sources

Our *C. maculata* colony originated from adults provided by colleagues at USDA facilities in Brookings, SD and Beltsville, MD, USA. Each life stage (eggs, larvae, pupae or adults) was reared in separate containers in a climate-controlled room (24 °C, 50%–60% RH, 16 h photophase). This colony has been maintained without any introduction of “wild” individuals for over 20 consecutive generations. Larvae and adults have been reared on eggs of the Mediterranean flour moth *Ephestia kuehniella* (*E. kuehniella*) Zeller (Lepidoptera: Pyralidae). Frozen-fresh *E. kuehniella* eggs were purchased from Beneficial Insectary Inc. (Redding, California, USA) at a cost of $ 3.80 USD per 28.35 g. Upon arrival, eggs were stored in a lab refrigerator (at −20 °C), until ready for use.

### 2.2. Rearing Density

*C. maculata* eggs were harvested at random from several *C. maculata* mating pairs, approximately 1-month old adults, from our rearing colony and assigned randomly to Petri dish arenas. Rearing densities were 1, 5, 10, 15 and 20 *C. maculata* first instars per Petri dish (2.5 cm high, 9.0 cm diam., 159 cm^3^). We used three replicate Petri dish arenas per treatment density in three identical trials, for a total of 45 arenas. There were 153 *C. maculata* first instars at the onset of each trial, for a total of 459 individuals for the entire experiment (three trials). A powdered formulation of brine shrimp *Artemia franciscana (A. franciscana)* Kellogg eggs (Anostraca: Artemiidae) [33] was used as food in each treatment arena. Freeze-dried, decapsulated *A. franciscana* eggs were purchased from Brine Shrimp Direct Inc. (Ogden, Utah, USA) at a cost of $ 0.99 USD per 28.35 g [15]. Each arena was provisioned with treatment food and covered with a mesh-screened lid. The quantity of food in each arena exceeded the amount that *C. maculata*, collectively, could consume in two days. Food was replaced, and uneaten food discarded, every other day. Each arena was provisioned with a small wad of cotton moistened with distilled water to provide a source of water. No refuges (hiding places) were provided in the arenas. Collected data included development time, larval and pupal survival rate, proportional mortality amongst larval instars, adult emergence rate, body size (live weight of teneral males and females), and sex ratio (*i.e.*, percentage of females emerging from pupae). All experimental arenas were maintained in a growth chamber (24 °C, 60% RH, 16 h photophase), but removed from the chamber each day to record life history (growth and development) data, replenish food, and re-moisten cotton wads as needed.

### 2.3. Statistical Analysis

Data on survival, growth, and development were averaged across replicate Petri dish arenas; the arena represented the sampling unit. Data on mortality amongst larval instars were not analyzed. Data on adult body weight were averaged across adults; the adult represented the sampling unit. Initially, all data were analyzed following a completely randomized design and absolute data and percentage data were square root transformed and arcsine transformed, respectively, if the assumptions of equal variance and normality were not met [34]. Despite transformation, survival, growth, and development data failed to meet the assumptions of normality; the non-parametric Kruskal-Wallis analysis of variance (K-W ANOVA) with *H* statistic was used for data analysis. The one-way analysis of variance (1-way ANOVA), using Standard Least Squares with *F* statistic, tested the effects of rearing density on female and male body weight. When necessary, the Steel-Dwass test or Tukey-Kramer HSD test was used as a multiple comparison procedure after the K-W ANOVA or 1-way ANOVA, respectively. JMP^®^ 10.0.0 (2012 SAS Institute Inc., Cary, NC, USA) and SigmaStat 3.0.1 (interfaced through Sigma Plot 12, Systat Software Inc., Richmond, CA, USA) software assisted with analysis of data.

## 3. Results

**Figure 1:**
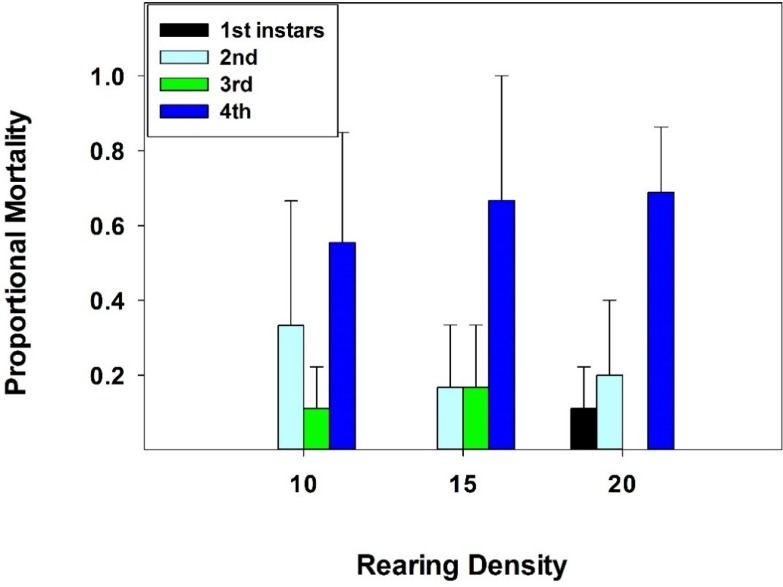
Mean ± SE proportion of dead 1st–4th instar larvae in 10, 15, and 20 density treatments in replicate trials. A total of 33 dead larvae were observed.

Rearing density had a significant effect on larval survival rate (*H* = 19.9; df = 4; *p* < 0.001; K-W ANOVA; *n* = 45 arenas). Slightly more larvae survived at the 1 density than the 10, 15, and 20 densities, and the 5 density than the 20 density (Table 1), but no differences were evident between the 10, 15, and 20 densities. All larvae at the 1 density treatment survived in all arenas, in all trials. Larval survival never dropped below 80% at any other density in the three trials. When mortality did occur, late 4th instars appeared to suffer the most (Figure 1). The dead larvae-in decreasing order of mortality-were 4th instars, 2nd instars, 3rd instars, and 1st instars, averaged over three trials, with a total of 8, 15, and 10 dead larvae in the first, second, and third trial, respectively.

**Table 1:** Median values and confidence intervals for pre-imaginal survival, development time, and sex ratio of teneral adults in relation to rearing density.

Life Parameter	Rearing Density	Median	10% C.I.	25% C.I.	75% C.I.	90% C.I.
Larval Survival (%)	1	100 ^a^	100	100	100	100
	5	100 ^ab^	80.0	100	100	100
	10	90.0 ^bc^	80.0	85.0	95.0	100
	15	93.3 ^bc^	80.0	88.3	100	95
	20	95.0 ^c^	85.0	92.5	95.0	100
Time as Larva (days)	1	14.0 ^a^	11.0	11.5	14.0	14.0
	5	13.0 ^a^	11.8	12.1	13.8	14.0
	10	13.7 ^a^	11.8	12.3	14.0	14.4
	15	13.6 ^a^	12.8	13.1	14.0	14.2
	20	13.2 ^a^	12.0	12.2	13.8	14.0
Time as Pupa (days)	1	3.0 ^a^	2.0	2.0	4.0	5.0
	5	4.0 ^a^	3.6	3.9	4.9	5.0
	10	3.9 ^a^	3.2	3.4	4.4	4.7
	15	3.8 ^a^	3.5	3.7	4.2	4.5
	20	4.0 ^a^	3.6	3.8	4.7	4.7
Pupal Survival (%)	1	100	100	100	100	100
	5	100	100	100	100	100
	10	100	100	100	100	100
	15	100	100	100	100	100
	20	100	100	100	100	100
Sex Ratio (% ♀♀)	1	100 ^a^	0	0	100	100
	5	50.0 ^a^	40.0	40.0	60.0	80.0
	10	55.6 ^a^	33.3	47.2	64.6	66.7
	15	50.0 ^a^	42.9	46.7	53.6	53.8
	20	52.6 ^a^	42.1	45.7	55.3	63.2

Median values followed by a different letter in a column, for each life parameter, are significantly different (*p* < 0.05, Steel-Dwass test). Sample size, *n*, 9 arenas per density. Rearing density represented number of larvae per arena (Petri dish, 2.5 cm high, 9 cm diam., 159 cm^3^).

**Figure 2:**
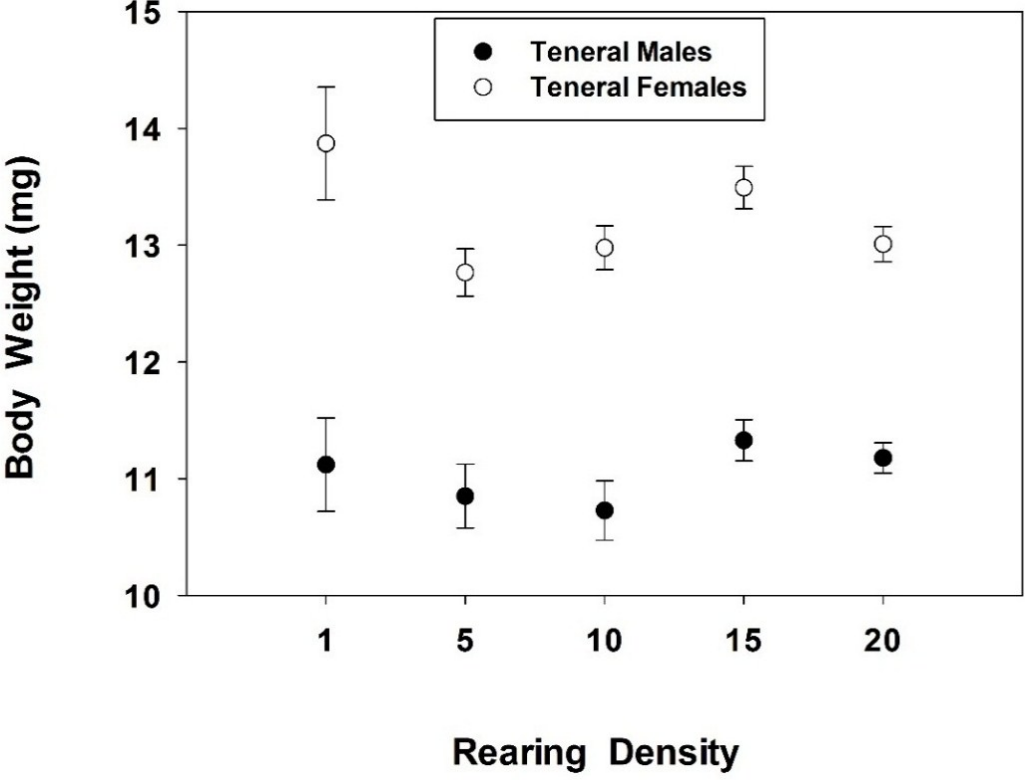
Mean ± SE body weight of teneral adults in relation to rearing density. A total of 208 males and 221 females were in the analysis.

Larval development time was unaffected by rearing density (*H* = 2.7; df = 4; *p* = 0.61; *n* = 45); neither was the time from the pupal stage to the teneral adult (*H* = 6.1; df = 4; *p* = 0.19; *n* = 45; Table 1). Rearing density had no effect on pupal survival rate (since all pupae emerged as adults) or sex ratio, *i.e.*, percentage of females (*H* = 1.2; df = 4; *p* = 0.87; *n* = 45). A total of 221 females and 208 males emerged from pupae in the combined trials, regardless of density treatment. The breakdown was 5, 23, 44, 63, and 86 females in the 1, 5, 10, 15, and 20 density treatments; 4, 21, 37, 64, and 82 males in the 1, 5, 10, 15, and 20 density treatments, respectively. Rearing density did not significantly affect body weight of teneral females (*F* = 2.28; df = 4, 216; *p* = 0.061) or teneral males (*F* = 1.46; df = 4, 203; *p* = 0.21) in this experiment (Figure 2).

## 4. Discussion

The hypothesis that a low to moderate rearing density has limited or no effects on pre-imaginal survival of *C. maculata* was partially confirmed in this study. A median survival rate of 90% for larvae at the highest densities (10, 15, and 20 larvae/159 cm^3^), suggests very limited negative effects. When mortality did occur, late 4th instar *C. maculata* larvae, which were partially immobile as they began to metamorphose into prepupae, died more often, presumably from being partially eaten by slightly younger larvae. Developing coccinellid larvae are more vulnerable to attacks from sibling larvae when molting, because they are motionless [35]. Cannibalism is an impediment to mass producing coccinellids for biological control [7]. Techniques to reduce larval-larval encounters, thereby reducing cannibalism on motionless (*i.e.*, molting) individuals could involve provisioning arenas with refuges or hiding places for molting or for pupation. Provisioning arenas with refuges or hiding places might have allowed an even higher *C. maculata* rearing density in this study. Although refuges or hiding places were not provided, a few previous studies have documented this technique. For example, researchers have used corrugated paper or plastics in 12-liter containers to create refuges to increase survival rate of the coccinellid *Lemnia biplagiata* (Swartz) [30]. Others have used excelsior (wood shavings) as refuges in 1-liter glass jars to reduce bodily contact between *Coccinella septempunctata* L. larvae, thereby increasing survival rate [31]. The observation that 100% of pupae metamorphosed into adults, regardless of rearing density, was not unusual, since over 95% of *C. maculata* pupae survived to the teneral adult stage, when reared in groups, in previous works [14,15,16]. Likewise, the observation that rearing density did not affect development time and sex ratio of emerging adults, in this study, further suggests that the moderate rearing densities (10, 15, and 20 larvae) had only minor, negative effects on *C. maculata* growth and development. Development time and sex ratio of teneral adults of the coccinellid *Brumoides suturalis* (F.) did not differ significantly between rearing densities of 1 *vs.* 10 larvae per arena [26].

One purpose of this study was to establish a suitable rearing density for *C. maculata* from the standpoint of developing space-efficient rearing methods applicable to mass rearing of this predator at a commercial scale. In several of our previous works [14,15,16], we designed experiments to test the suitability of alternate foods for *C. maculata*, when reared in groups of 10–12 larvae per arena (159 cm^3^). Several of our critics commented that we should have reared larvae individually instead. From our standpoint of developing efficient rearing techniques, rearing larvae individually is not acceptable. Hence, an underlying reason for conducting this study, and for limiting the experimental design to include low to moderate rearing densities (rather than high rearing densities), was simply to validate that group rearing of *C. maculata* larvae was possible. This study was not designed to determine the maximum density of *C. maculata* larvae that could be packed into a 159 cm^3^ arena. However, we are aware that *C. maculata* can be reared successfully at a density of 30–40 larvae per 159 cm^3^ when fed powdered *A. franciscana* eggs (Riddick, E.W. and Wu, Z., unpublished data [36]).

Based on the data generated in this study, a rearing density of 20 larvae per arena (159 cm^3^) would ensure successful rearing. We define successful rearing as the capacity to rear 70% or more larvae to the adult stage per arena. The 20 larvae/159 cm^3^ can be converted into 0.126 larvae/cm^3^, to provide a means of comparing successful rearing densities of other predatory insects (see Table 2). We propose that the greater the successful rearing density, the greater the potential of rearing on a commercial scale of production. We calculated the successful rearing density of several ladybird species, known as predators of aphids in agroecosystems, including *B. suturalis*, *Adalia bipunctata* (L.), and *Propylea dissecta* (Mulsant) (see Table 2). A rearing density of 1 *versus* 10 larvae of the three-striped ladybird *B. suturalis*, in Petri dish arenas (1.5 cm high, 14.0 cm diameter) provisioned with the aphid *Aphis gossypii* Glover, had no effect on pre-imaginal survival; approximately 80% of larvae metamorphosed into pupae and 90% of pupae metamorphosed into adults at either density [26]. Using 10 larvae per arena (230.79 cm^3^), the successful rearing density was 0.043 larvae/cm^3^ for *B. suturalis*. Larvae of *A. bipunctata* were reared at densities of 1, 2, 4, 8, 16, and 32 individuals in glass jars (20 cm high, 15 cm diameter) and fed the aphid *Myzus persicae* (Sulzer) [24]. A rearing density of 4 larvae per arena ensured a survival rate of 75%, which converts into a successful rearing density of 4 larvae/3,532 cm^3^ or 0.0011 larvae/cm^3^ for *A. bipunctata* (see Table 2). Larvae of *P. dissecta* were reared in plastic beakers (11 cm high, 9 cm wide) at densities of 1, 4, 8, 16, 25, and 35 larvae per beaker and fed the aphid *Aphis craccivora* Koch [25]. Rearing at a density of 25 larvae per beaker resulted in 88% larval survival, but body weight of the resultant adults (6.2 mg) was significantly less than those reared at a density of 4 larvae per beaker (*i.e.*, 11.2 mg). Consequently, the authors concluded that 4 larvae per beaker was the most successful rearing density. Therefore, 4 larvae/699.43 cm^3^ converts into a successful rearing density of 0.006 larvae/cm^3^ (Table 2).

**Table 2:** Comparison of larval density ensuring a survival rate of 70% or greater, successful rearing density, and female body weight of ladybird beetles used in biological control.

Predator	Larvae per Arena at 70%+ Survival Rate *	Arena Height (h) × Diameter (d), in cm	Arena Volume (cm^3^) ^1^	Successful Rearing Density (Larvae/cm^3^)	Body Weight (mg), at 70%+ *	Reference
*Coleomegilla maculata*	20	2.5 × 9.0	158.96	0.126	13.0	This study
*Brumoides suturalis*	10	1.5 × 14.0	230.79	0.043	-	[26]
*Adalia bipunctata*	4	20 × 15	3,532	0.001	-	[24]
*Propylea dissecta*	4	11 × 9	699.43	0.006	11.2	[25]

* The number of ladybird larvae per arena demonstrating a survival rate of at least 70%. ^1^ Arena volume was determined from the equation for the volume of a cylinder [*V* = π *r*^2^ *h*]. The “successful rearing density” is an estimate of the ladybird larvae/cm^3^ with a survival rate of at least 70%. Recognition of female body weight of reared predators can be factored into the successful rearing density to more accurately compare rearing density amongst species, assuming that body weight (mass) is directly proportional to body size (length × width).

Clearly, successful rearing density is greater for *C. maculata* than the other coccinellids and body weight can factor into successful rearing density (0.126 larvae/cm^3^ × 13.0 mg/adult = 1.64 mg/cm^3^). Body weight provides an estimate of body size (length and width); larger sized species may or may not make more bodily contact with conspecifics than smaller sized species in the confines of a shared arena.

The reasons why *C. maculata* larvae are more amenable to group rearing than the other coccinellids mentioned in this study could reflect diet preferences. Although *C. maculata* larvae prefer aphids, they can complete development on a plant-based diet [8,9,16], which suggests that requirements for nutrients from animal (invertebrate) sources are less stringent, in comparison to other species not known to complete development on plant products. Therefore, developing *C. maculata* larvae do not need to cannibalize sibling larvae to glean nutrients not found in suboptimal foods. That being stated, this study highlights the suitability of powdered *A. franciscana* eggs for *C. maculata* development. *Adalia bipunctata* larvae are rarely capable of developing successfully on a plant-based diet, such as moist bee pollen [37]. The ability of either *B. suturalis* or *P. dissecta* larvae to develop on pollen, or any other plant product, is unreported in the literature to our knowledge, suggesting that both species are distinctly carnivorous [7]. An alternative explanation for successful group rearing of *C. maculata* larvae could be related to behavior and artificial selection. The insects used in this study originated from a colony adapted to group rearing. Therefore, we can’t rule-out the possibility that lab-adaptation had some underlying influence on the propensity of larvae in our experiment to tolerate rearing in close quarters with conspecifics. Further research could test for differential effects of rearing density on lab-adapted *versus* wild (feral) *C. maculata* larvae.

## 5. Conclusions

In this study, more larvae survived at the 1 and 5 densities, but no differences were detected between the 10, 15, or 20 densities. Nevertheless, median survival rate was 90% or greater for larvae and 100% for pupae at the 10, 15, and 20 densities. Development time, body weight, and sex ratio were unaffected by rearing density. Overall, this study suggests that *C. maculata* larvae can be mass produced successfully at a density of 20 larvae/159 cm^3^ (≈0.126 larvae/cm^3^) in containers provisioned with powdered *A. franciscana* eggs.

Scale-up of rearing systems from experimental arenas to rearing containers is necessary to reach the capacity for inundative releases [38]. Clearly, we would need to scale-up the rearing of *C. maculata* from 159 cm^3^ to maybe 5000 or 10,000 cm^3^ containers to facilitate commercial scale rearing. Since 85%–95% of 20 *C. maculata* larvae were successfully reared in 159 cm^3^ arenas, we predict that at least 85% of 629 larvae can be reared in 5,000 cm^3^ containers (*i.e.*, 20/159 cm^3^ ≈ 629/5000 cm^3^). Future research on space-efficient rearing of *C. maculata* should focus at the commercial scale. Based on the meager literature on rearing density of predatory insects, along with the results of this study, we surmise that *C. maculata* has considerable rearing capacity and promise for commercial production for augmentative biological control.
